# Solar‐Driven Soft Robots

**DOI:** 10.1002/advs.202004235

**Published:** 2021-02-22

**Authors:** Seyed M. Mirvakili, Arny Leroy, Douglas Sim, Evelyn N. Wang

**Affiliations:** ^1^ Koch Institute Massachusetts Institute of Technology Cambridge MA 02139 USA; ^2^ Mechanical Engineering Massachusetts Institute of Technology Cambridge MA 02139 USA; ^3^ Electrical and Computer Engineering Department University of British Columbia Vancouver BC V6T 1Z2 Canada

**Keywords:** actuators, artificial muscles, phase change, soft robots, solar energy, untethered

## Abstract

Stimuli‐responsive materials have been lately employed in soft robotics enabling new classes of robots that can emulate biological systems. The untethered operation of soft materials with high power light, magnetic field, and electric field has been previously demonstrated. While electric and magnetic fields can be stimulants for untethered actuation, their rapid decay as a function of distance limits their efficacy for long‐range operations. In contrast, light—in the form of sunlight or collimated from an artificial source (e.g., laser, Xenon lamps)—does not decay rapidly, making it suitable for long‐range excitation of untethered soft robots. In this work, an approach to harnessing sunlight for the untethered operation of soft robots is presented. By employing a selective solar absorber film and a low‐boiling point (34 °C) fluid, light‐operated soft robotic grippers are demonstrated, grasping and lifting objects almost 25 times the mass of the fluid in a controllable fashion. The method addresses one of the salient challenges in the field of untethered soft robotics. It precludes the use of bulky peripheral components (e.g., compressors, valves, or pressurized gas tank) and enables the untethered long‐range operation of soft robots.

## Graphical abstract

While offering immense potential for disruptive applications, pneumatically driven soft robots require bulky, heavy, and pricy peripherals, such as gas tanks, compressors, and valves, to operate. Here, a technique to fabricate untethered soft robots that are directly powered by solar energy is presented.

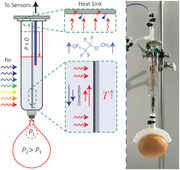

## 1 Introduction

Inspired by nature, researchers have developed soft robots that can perform complex robotic maneuvers, emulating biological systems, with their compliant structure.^[^ ^1^ ^]^ Unlike rigid robots, where only six degrees of freedom exist—due to the compliant structure of soft robots—they can offer an infinite number of degrees of freedom (e.g., torsion, bending, elongation, compression, wrinkling, buckling).^[^ ^2^ ^]^ This large number of degrees of freedom allows shape‐invariant and pose‐invariant grasping with simple control schemes.^[^ ^3^ ^]^

To drive soft robots, muscle‐like actuators (artificial muscles) have been employed in the form of tendon‐like fiber actuators (e.g., shape memory alloys, nylon fibers) or fluid‐driven actuators (e.g., pneumatic, hydraulic).^[^ ^1^, ^4^ ^]^ Pneumatic actuators (pneumatic artificial muscles, or PAMs) generate strains and stresses very comparable to those of human skeletal muscles while exhibiting a simple actuation mechanism.^[^ ^5^ ^]^ One of the fundamental limitations of PAM‐based soft robots is the size/weight requirement of valves, compressors, and pumps.^[^ ^6^ ^]^ Other mechanisms to generate the required pressure for actuation such as combustion,^[^ ^7^ ^]^ gas evolution reactions,^[^ ^8^, ^9^ ^]^ chemically activating swelling/deswelling,^[^ ^10^, ^11^ ^]^ and phase change materials^[^ ^12^, ^13^, ^14^, ^15^, ^16^ ^]^ have been explored. Except for phase‐change materials, the rest of the mentioned techniques are irreversible processes and cannot be used for the continuous and prolonged operation of the soft robots.^[^ ^16^ ^]^

Heat, generated by various mechanisms (e.g., light, magnetic induction, or Joule heating), is the driving force for thermal actuators.^[^ ^5^, ^17^, ^18^ ^]^ Light‐induced thermal actuation or photo‐thermal actuation has been explored extensively with thermo‐responsive polymers,^[^ ^19^ ^]^ liquid crystal elastomers,^[^ ^20^, ^21^ ^]^ hydrogels doped with light‐absorbing materials,^[^ ^22^, ^23^, ^24^, ^25^ ^]^ and silicon microcantilevers for atomic force microscopy.^[^ ^5^ ^]^ While these materials offer some forms of actuation for small scale devices, they operate with high energy photons in the UV spectrum or high intensity infrared light generating temperatures >150 °C making them not suitable for driving soft robots to perform untethered manipulation or locomotion at larger scales. Other types of nonthermal light‐induced actuation mechanisms such as photostriction,^[^ ^5^ ^]^ *cis*–*trans* photoisomerization in azobenzenes,^[^ ^5^ ^]^ photomechanical elastomers,^[^ ^26^ ^]^ and photoreversible [2+2] cycloaddition reactions in polymers containing cinnamic groups exist.^[^ ^27^ ^]^ Nonetheless, they operate with UV light—with exposure time of more than an hour, exhibit slow response time and small strain/stress not sufficient for realization in high performance soft robotic embodiments.

In this work, we propose a low‐cost, highly functional, and easy‐to‐implement technique for driving untethered soft robots. The method involves generating high‐pressure gas via light‐induced evaporation of a low‐boiling point liquid confined in a chamber. The relatively large volumetric expansion in the liquid‐to‐gas phase transition (e.g., 1600 for water at standard conditions) is harnessed to actuate soft robotic prototypes hydraulically. Thus, the actuation mechanism can be recognized as an untethered pneumatic–hydraulic hybrid actuation.

With our proposed technique, we demonstrate untethered soft robotic grippers grasping and lifting objects almost 25 times the mass of the phase‐change fluid when exposed to white light. We implemented our design with readily available components with a total cost of $68 (USD) for one unit. Owing to its simplicity, the design can be downscaled easily for application in miniature soft robotics. Unlike electric and pneumatic sources, sunlight is available in most parts of the planet and the solar system making our design widely applicable for a variety of applications.

## 2 Results and Discussion

**Figure 1:**
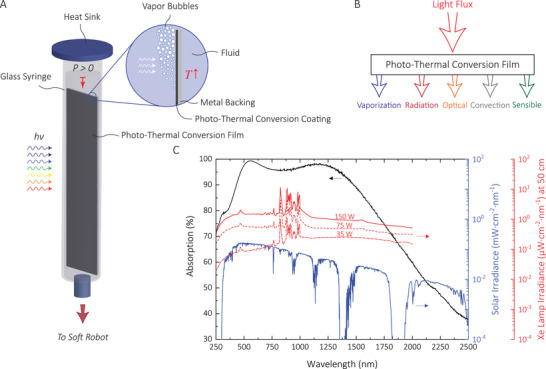
A) Making of a light‐driven soft robot pressure chamber. For visual illustration purposes, the evaporation process is shown with vapor “bubbles.” B) The energy accounting of the photo‐thermal conversion film. C) The absorption spectrum of the selective solar absorber film. Spectral power density for the solar radiation and Xe lamps with an input power of 35, 75, and 150 W (courtesy of Hamamatsu Photonics K.K.).

The device comprises two major parts: the pressure chamber (containing the low‐boiling point liquid (34° at 1 atm) and the solar absorber film) and the soft robot prototypes (made of compliant silicone rubber). The pressure chamber is made of a glass syringe with its one end sealed with a heat sink, and the other end connected to a soft robot (**Figure** **1**A). To accelerate the evaporation of the low‐boiling point liquid under light irradiation, we used a commercially available selective solar absorber film (Experimental Section). These films absorb sunlight with absorptance of more than 95% in the visible range.^[^ ^28^ ^]^ Nanomaterials such as carbon nanotubes,^[^ ^29^, ^30^ ^]^ MXenes,^[^ ^31^ ^]^ and graphene^[^ ^32^ ^]^ offer better solar absorptance—more than 99% for Vantablack—but their current production cost makes them an unfeasible solution for integration in soft robots.

When exposed to a sufficiently high‐intensity white light, the film heats the fluid, which significantly increases its vapor pressure and thus its evaporation. However, as the fluid and chamber heat up above the ambient temperature, heat losses to the environment (Figure 1B) also occur, which reduces the temperature rising rate in the chamber. These heat losses are, however, necessary for a fast relaxation (cooldown of the fluid and thus drop in pressure) of the soft robot from the excited state. Optimization of the solar heat gains and heat loss mechanisms, therefore, play a crucial role in increasing the actuation rate.

To best simulate the solar light spectrum in a more controlled indoor environment, we employed a Xenon lamp (55 W) (Figure 1C) (see the Experimental Section; Supporting Information). From the spectral irradiance profile, we found the irradiance of the Xe lamp to be 6.85 W m^−2^ at 50 cm (Supporting Information). To achieve a higher intensity, we employed a reflector (Experimental Section), enabling irradiance more than 4 kW m^−2^ (Supporting Information). For outdoor tests, we performed the experiments under sunlight with an estimated power of 1 ± 0.2 kW m^−2^ (Experimental Section).

Due to the complexity of the heat transfer and kinetics of the system, we developed a simple 1D analytical model to understand the system better and evaluate the influence of system parameters (Modeling, Supporting Information). We used this model to estimate the time‐dependent temperature and pressure of the pressure chamber as a function of the input light flux. The model couples the first law of thermodynamic around the pressure chamber with the ideal gas law in the vapor gap, and conservation of mass to the fluid inside the pressure chamber to solve for the system's three unknowns—system's temperature *T*, the mass of fluid in the liquid (*m*_f_) and gaseous (*m*_g_) state (Modeling, Supporting Information). We validated our analytical model with a 3D COMSOL model of the system. More details of the analytical and numerical models are given in the Supporting Information. While our numerical simulation predicts the system more accurately by accounting for natural convection and temperature gradients within the fluid and gas, it is computationally intensive and takes longer (hours vs seconds) to converge compared to the analytical model. Thus, we used the analytical model to study the system.

**Figure 2:**
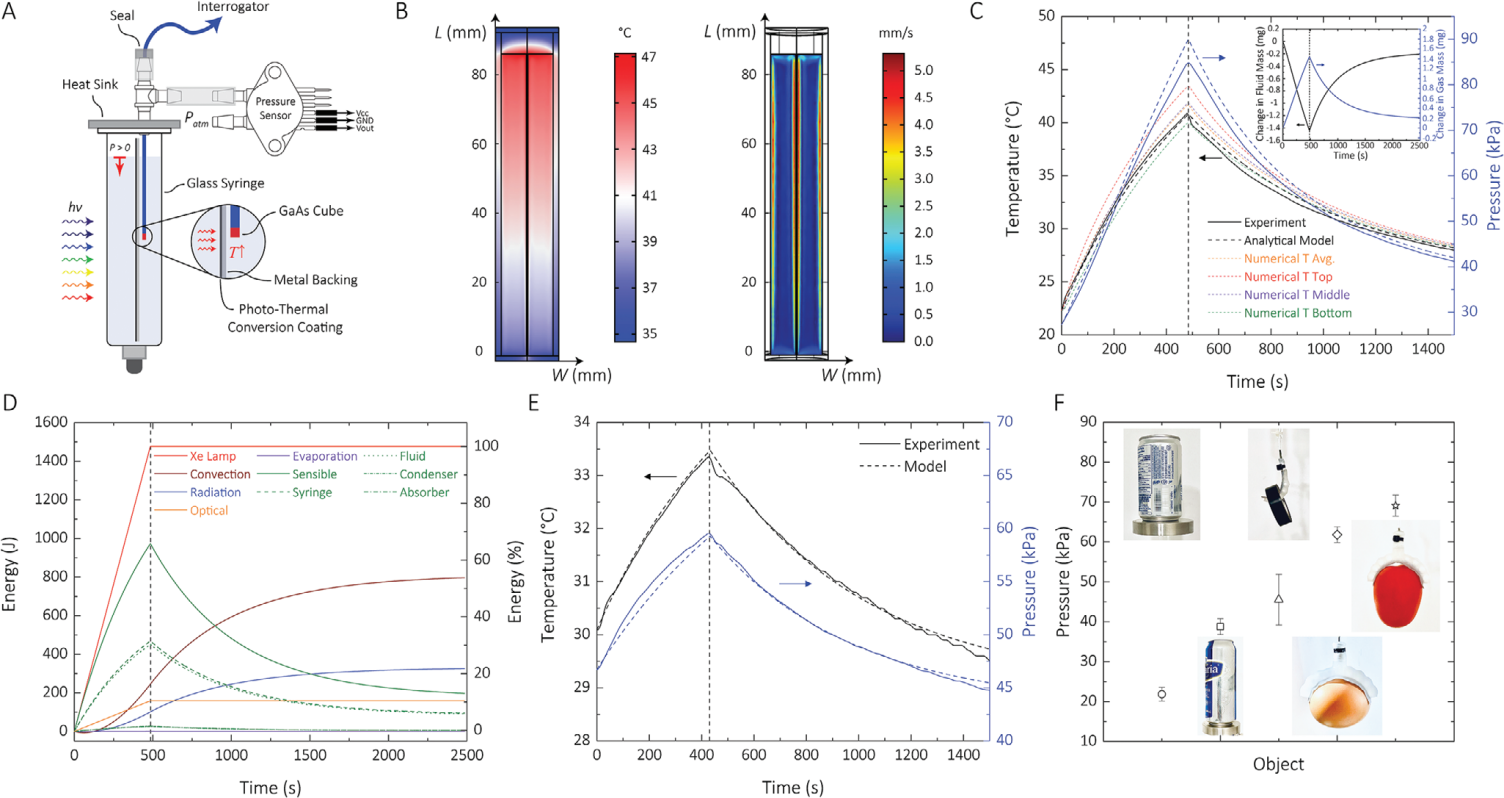
A) Illustration of the setup for the closed‐system experiments. The optical fiber temperature sensor is illustrated in blue with its GaAs tip in red. To sense the average temperature of the fluid in the syringe, we placed the temperature probe close to the backside of the film in the middle of the syringe. The pressure sensor is connected to a microcontroller. The illustration is not to scale. B) Snapshots of the numerical simulation of the system with 14 mL of fluid under the illumination of 2450 W m−^2^ for 480 s. Left: 2D map of the temperature, right: 2D map of the convection in the syringe. C) The analytical model predicting the behavior of the system in (B). The analytical model, numerical model, and the experimental data for the pressure and the temperature show a close fit. The Numerical T Top/Bottom are evaluated at 2.15 cm above and below the centerline, respectively. Inset: The change in fluid and vapor mass during the excitation. D) The energy accounting for the system. Sensible heating consists of the sensible heat of the syringe, fluid, condenser, and absorber. E) A closed system with a 7 mm air gap excited with indirect sunlight (indoor) with a power density of 630 W m^−2^. F) The pressure required to lift different objects. The first two objects with masses of 236 and 523 g (from left) were lifted by a hydraulic glass syringe while the rest are lifted by the soft robotic grippers. The mass of the barrel (14.65 mm in diameter) and the metallic plate was 119 g. The black tape (14 g) was lifted by a soft robotic arm. The egg (62 g) and the tomato (86 g) were lifted by a soft robotic gripper.

To evaluate the model, we first performed closed‐system experiments without soft robots. One differential pressure sensor and one temperature sensor were incorporated in the setup to measure the experimental parameters (Experimental Sections) (**Figure** **2**A). The measured temperature in the middle of the syringe was compared with our numerical model results (Supporting Information). As illustrated in Figure 2B,C, the average temperature suggested by the simulation closely matches the measured temperature in the center of the chamber. Our numerical simulation showed that during the excitation periods, a convective flow occurs at a speed of about 5 mm s^−1^ at both surfaces of the film (Figure 2B). All these convective flows transfer heat across the system and reduce the thermal gradients in the syringe (Supporting Information).

Our detailed quantitative analysis of the energy accounting for the closed‐system revealed that sensible heating accounts for 65.9% of the total energy received by the film (Figure 2D). The sensible heating includes heating of the fluid, the condenser (i.e., heat sink), the syringe, and the absorber film. The results show that sensible heating for the syringe is almost equal to that of the fluid, both accounting for most of the absorbed solar flux. The heat capacity of the system, in this case, dominated by the fluid and the syringe, plays a significant role in determining how fast the system can heat up and cool down. Decreasing the mass or specific heat of the fluid or the syringe could significantly help to reduce the heating and cooling time for faster actuation times. Acrylic is transparent and machinable but possesses specific heat capacity higher than that of the glass (2.16 J g^−1^ K^−1^ vs 0.83 J g^−1^⋅K^−1^). Reducing the thickness of the glass syringe wall can help to minimize the syringe sensible heating. Other losses, such as convection, radiation, and optical losses (i.e., glass transmittance), account for 16.57%, 6.84%, and 10.76% of the total energy balance, respectively. Thermal losses are necessary to achieve fast cooling and thus actuation of the device. Increasing the effective heat transfer coefficient with the ambient using radiative cooling or extended surfaces (e.g., fins) can be used at the expense of higher heat losses during heating and increased thermal mass.

Evaporation energy accounts for only 0.014% of the total energy. This small portion corresponds to the small, evaporated mass of the fluid (≈1.5 mg) (Figure 2C inset), which is limited by the temperature‐dependent vapor pressure of the liquid and the free volume at the top. A similar closed‐system experiment was performed under sunlight (630 W m^−2^) in a closed environment (indoor—behind windows). Unlike excitation with the Xe lamp, the experiment conditions such as the ambient temperature and light intensity (e.g., clouds covering the sun) fluctuated during the experiment (Figure 2E).

The peak pressure (85 kPa) generated in the closed‐system experiments is enough to actuate robotic arms and linear pneumatic air cylinders to lift objects as heavy as 0.5 kg, 25 times the mass of the fluid in the syringe (Figure 2F). As shown in Figure 2F, the required pressure for grasping objects like an 86 g tomato is almost double than that for lifting a 642 g soda can (including the barrel and the plate). This disproportionality can be explained by the fact that the friction force for grasping an object is proportional to the normal force and the coefficient of friction. Therefore, the more slippery or smaller the coefficient of the friction at the surface of an object is, the higher the normal force needs to be to grasp on that object. With the peak pressure that we measured, in a hydraulic configuration, the device can be expected to lift a 1.3 kg object with only 15 mL of the phase‐change fluid. More prolonged light exposure can lead to higher pressures, thus, higher output forces.

**Figure 3:**
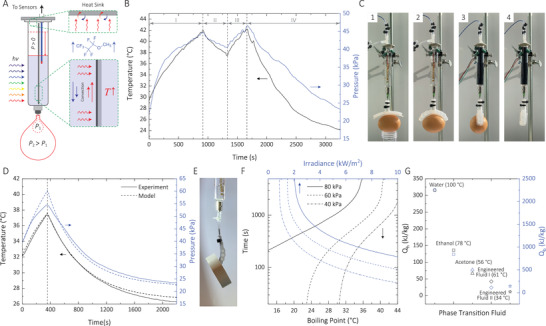
A) Setup for the light‐driven soft robots. B,C) Temperature and pressure profiles of a light‐driven soft robotic gripper grasping on a 60 g boiled egg excited with direct sunlight. The experiment was performed outside in a non‐controlled environment. During the period I the device heats up until it grasps the egg C1,2). To prevent the soft robotic gripper from bursting, we rotated the pressure chamber by 90° C3). In this case, period II, the actuator cools down until the egg slips C4). This process is repeated once in periods III and IV. D,E) Temperature and pressure profiles for a robotic arm holding a tape roll under Xe lamp irradiation. F) The time it takes to reach 40, 60, and 80 kPa of gauge pressure as a function of boiling point and solar irradiance. For both the boiling point and irradiance simulations, an initial temperature of 25 °C and partial pressure of air of 75 kPa. The initial temperature was 26.5 °C for the boiling point and irradiance simulations. The irradiance for the boiling point simulation was 1850 W m^−2^. G) The latent heat of evaporation and heat per mass to increase the temperature of five different liquids from 25 °C to the boiling point (written in by the name of each fluid).

To demonstrate the feasibility of our proposed design for integration with soft robotics, we fabricated two types of soft robot prototypes as exemplars—robotic grippers with two fingers and robotic arms (**Figure** **3**). The soft robots were connected to the pressure chamber with a blunt needle (Figure 3A). We actuated soft robots with direct sunlight in a noncontrolled environment (Figure 3B). Due to the fluctuation in the environmental conditions, the temperature and pressure profiles were not as smooth as those for indoor experiments with the Xe lamp. Studies have shown that clouds can reduce solar irradiance by up to 80%.^[^ ^33^ ^]^ The reduction in solar irradiance can increase the actuation period.

We demonstrated under direct sunlight (1 kW m^−2^), the soft robotic gripper grasped on a 56 g boiled egg. To prevent the bursting of the gripper, we rotated the glass syringe by 90°, when the pressure reached about 45 kPa, to cool down the device by reducing solar absorption at the absorber (Figure 3B,C). The syringe was rotated back to its initial angular position when the pressure reached 40 kPa. At the final stage, the syringe was cooled down at the 90° angular position. With a soft robotic arm excited with the Xe lamp with 1.8 kW m^−2^ light intensity, we could demonstrate the arm holding a 40 g tape roll during the excitation cycle. The robotic gripper is capable of shape‐invariant grasping. However, for large radius ring‐shaped items that are too large for the soft robotic grippers, the robotic arm works better.

Our analytical model, for the complete device with the soft robotic grippers, predicts the temperature and pressure responses (Figure 3D). We speculate that the slight mismatch at the peak pressure is possibly due to condensation at the heat sink (reducing the pressure), large thermal gradients in the chamber, slow diffusion kinetics in the vapor gap, or even nonlinear mechanics of the prototype around that pressure.

For agile soft robots, the actuation response time can be a rate‐limiting factor. Our analytical model predicts that the boiling point and excitation irradiation are two critical parameters in determining the actuation time response for a specific design (Figure 3F). We chose a chemically inert engineered fluid (i.e., methyl perfluoropropyl ether) with a boiling point of 34 °C to achieve a relatively fast actuation rate. Compared to water, the engineered fluid that we are using (labeled as engineered fluid II on Figure 3G) requires 27 times smaller heat per mass to reach the boiling point (1 at vapor pressure) (from 25 °C). Similarly, it has a latent heat of evaporation 16 times lower than that of water.

Depending on the ambient temperature, fluids of different boiling points can be used. For example, methoxy‐nonafluorobutane, an inert organic fluid (labeled as engineered fluid I on Figure 3G), has a boiling point of 61 °C. Both acetone and engineered fluid I are ideal for warm temperature environments. Ethanol and water are the next candidates for temperatures above 70 °C.

Downscaling the device can enhance the actuation rate but at the expense of undermining the useful output force and degrees of freedom. For a pneumatic architecture, the pressure can be increased with more vapor or with higher temperatures. However, in our hybrid pneumatic‐hydraulic architecture, a portion of the fluid volume is contributing to actuating the soft gripper, thus determining a minimum required volume of fluid for operation. For small scale applications where a small range of forces is needed, the scaling can be advantageous.

Control is an essential element in soft robotics.^[^ ^34^ ^]^ For tethered actuators, electrical power or pneumatic pressure is controlled directly by a closed‐loop circuit.^[^ ^5^ ^]^ In contrast, for untethered phase‐change pneumatic actuators and soft robots, the pressure is developed in response to an external stimulus. Therefore, controlling the pressure or volume is vital to prevent the bursting of the soft robot.^[^ ^18^ ^]^ In this work, we implemented a low‐level control scheme in the actuation space.

**Figure 4:**
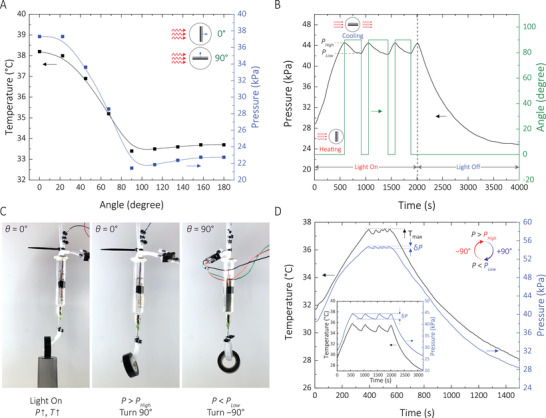
A) Temperature and pressure profiles as a function of the angular position of the syringe in a closed‐system experiment. B) Pressure profile for a soft robotic gripper (with no load) excited with a Xe lamp under 800 W m^−2^. When the pressure reaches *P*_High_, the stepper motor rotates by 90°. When the pressure reaches *P*_Low_ in the cooling cycle, the stepper motor rotates back to the initial angular position. Controller's output as a function of time and pressure. C) Snapshots of the robotic arm actuating with the control system activated. D) Temperature and pressure profiles for the experiment in (C). The *δP* is less than 1 kPa. Inset: Temperature and pressure profiles for the robotic gripper used in experiment (B).

As shown in **Figure** **4**A, the temperature and pressure of the system are functions of the light‐absorber film incident angle. By exploiting this relationship, we designed our control scheme to converge to the desired pressure by monitoring the pressure and modulating the incident angle (by rotating the glass syringe). Other proprioceptive sensors with alternative control schemes can be used to operate the soft robot smoothly. However, modulating the irradiation absorption precludes the use of exotic elements such as electrochromic films. Moreover, unlike shading—which is a binary effect—changing the angular position of the syringe offers a continuous spectrum of pressure/temperature values.

Rotating the glass syringe by 90° offers the largest drop in the temperature/pressure (Figure 4A). The temperature/pressure slightly increases above 90°, which is due to an increase in the exposed area of the film to the irradiation. At an angular position of 90°, light absorption by the glass syringe prevents a complete relaxation to room temperature, making it faster to reach the peak temperature and pressure in the next excitation cycle. This energy‐saving mechanism can be very advantageous for rapid “pick‐and‐place” maneuvers.

To implement the control scheme, we built a closed‐loop circuit in which the pressure is monitored continuously by a small microcontroller (Experimental Section, Supporting Information). When the pressure reaches *P*_high_ (set in the program), the microcontroller commands a small stepper motor to rotate the glass syringe by 90°. In this cycle, the temperature and pressure decrease until pressure reaches *P*_low_. At this point, the stepper motor rotates the glass syringe back to its initial angular position (Figure 4B). Therefore, the pressure always oscillates between *P*_high_ and *P*_low_. Using this control scheme, we could control the internal pressure of a robotic arm within only 1 kPa range. The arm could hold a tape roll for more than 200 s without collapsing (Figure 4C,D).

## 3 Applications

Due to the untethered nature of our proposed technique and the output force it can generate, it can be employed in a variety of real‐world applications. Aside from soft robotics, solar cells can also make use of the proposed actuation mechanism in their design. Solar cells best absorb the sunlight when the solar irradiation is at a specific angle with the surface of the panels. Due to the earth's rotation, the incident angle changes from the sunrise to the sunset. Currently, active solar trackers are mainly used to maintain the incident angle. Considering that the trackers consume energy to keep the incident angle constant, it is more efficient to utilize technologies that can achieve solar tracking passively. The sunlight or even the waste heat generated by solar cells can be harnessed to power up the actuator. Considering the slow actuation rate of the actuator, it can be very well suited for solar tracking applications. In a different scheme, the device can be used for space exploration applications by harnessing the ubiquitous light in the solar system.

## 4 Conclusion

We present a simple, low‐cost, and highly functional method for driving soft robots with light. The approach offers long‐range excitation of soft robots with collimated light or sunlight, enabling its application in places where electric or pneumatic sources are not available. With two different soft robot prototypes and a low‐level control scheme, we demonstrate lifting and grasping objects used in daily life. These capabilities suggest new possibilities for applications for soft robots and even rigid robots. Furthermore, our proposed technique can be implemented in scales (from nano to macro) in which implementing tethered soft robots is impossible.

## 5 Experimental Section

##### Pressure Chamber Fabrication

The body of the pressure glass was fabricated from a 10 mL borosilicate glass metal Luer–Lock syringe (Wheaton Science Products). After cleaning the inner surfaces of the glass syringe with 70% ethyl alcohol, a 14 mm × 88 mm piece of solar absorber film (eta plus AL) with a thickness of 0.4 mm was placed in the center of the syringe. The top end of the syringe was then sealed with a metallic film with a thickness of 1.9 mm and a diameter of 29.3 mm. A barbed tee connector (for 1/8” tube ID, McMaster‐Carr) was used to incorporate the temperature sensor fiber to the pressure chamber. For the closed‐system experiments, a Luer–Lock syringe tip cap was locked to the needle adapter to seal the syringe completely. For integration with soft robots, a blunt tip needle (15G–1.8 mm tip diameter) was used to connect the syringe to the soft robots. The connection point was reinforced with a zip tie.

##### Measurement and Control

The right‐angle branch of the tee connector was connected to a pressure sensor (MPX4250DP, 250 kPa differential) via a PVC plastic tube (ID 1/8” and OD 1/4”) (Figure 2A). A fiber‐optic temperature sensor (Optocon FOTEMP1‐OEM) was used to measure and log the temperature profile during actuation. An Arduino Mega 2560 microcontroller was employed to control and monitor/log the pressure. A 5V 4‐phase small stepper motor (Neuftech 28BYJ‐48) with a driver board (ULN2003 module for Arduino) was used with a control scheme (Figure 4B) to regulate the pressure in the system.

##### Excitation Setup

For indoor experiments, a 55W Xenon lamp (WinPower 55W 9012 8000K Xenon HID headlight) was utilized with its associated 12V ballast. To increase the light intensity, an A8 high beam reflector was used for Xe headlights (Supporting Information). Xenon lamps generated an irradiance spectrum with a similar profile as to the solar spectrum. Xenon‐Mercury arc lamps generated an almost flat spectral irradiance (Supporting Information), but unlike Xe lamps were expensive and not readily available. Outdoor experiments were performed under sunlight on sunny days with some light clouds occasionally covering the sun.

##### Soft Robotic Parts

The soft robotic pieces were fabricated through a previously reported molding process.^[^ ^18^ ^]^ In brief, the molds for the gripper and arm were 3D printed with a fused deposition modeling 3D printer (FlashForge Creator Pro) with polylactic acid thermoset filament (1.75 mm in diameter) and layer and print resolutions of 0.1 and 0.2 mm, respectively. EcoFlex 00–50 platinum‐catalyzed silicone rubber was used to fabricate the soft robotic parts. A one‐to‐one ratio of the two precursors for EcoFlex 00–50 was mixed and then degassed in a desiccator for 5 min with a rotary vacuum pump. After filling the molds with the mixture, they were degassed further and cured at the room temperature for 4 h. A piece of cotton fabric was adhered to the gripping side of the soft robot by coating the fabric with EcoFlex 00–50. This step was essential to create an anisotropic expansion of the soft robot upon excitation produce bending.

##### Engineered Fluids

Engineered fluid I and II refer to methoxy‐nonafluorobutane and methyl perfluoropropyl ether which are commercially available under 3M Novec 7100 and 3M Novec 7000, respectively.

##### Cost

The total cost of the components used in this device was estimated including the electronics, the glass syringe, solar absorber film, the low‐boiling point fluid, the soft robotic pieces, and other pieces (e.g., connectors, batteries, etc.) to be $68 (USD).

##### Statistical Analysis

The numbers that are expressed in the format of mean ± SD are calculated from six repeated measurements.

## Conflict of Interest

The authors declare no conflict of interest.

## Supporting information

Supporting Information

Supplemental Video 1

Supplemental Video 2

## Data Availability

Data available on request from the authors.
