# Supplementary material for: Solar‐Driven Soft Robots
Source: Adv Sci (Weinh). 2021 Feb 22;8(8):2004235. doi: 10.1002/advs.202004235 (PMC8061385; doi:10.1002/advs.202004235)
Supplement: Supplementary file 1 — Supporting Information [file ADVS-8-2004235-s003.pdf]

© 2021 Wiley-VCH GmbH

ADVANCED SCIENCE
Open Access

Supporting Information

for *Adv. Sci.,* DOI: 10.1002/advs.202004235

Light-Driven Soft Robots

Seyed M Mirvakili*, Arny Leroy, Douglas Sim, Evelyn N. Wang

Supporting Information for
Light-Driven Soft Robots

Seyed M Mirvakili[1,*], Arny Leroy[1], Douglas Sim [2], Evelyn N. Wang [1]

[1]Massachusetts Institute of Technology, Cambridge, MA 02139, USA.

[2]University of British Columbia, BC, Canada.

*Corresponding to: seyed@mit.edu

This PDF file includes:

- Additional Experimental Information
- Control Implementation
- Modeling
- Figures S1 and S4
- Reference S1

Other Supplementary Material for this manuscript includes the following:

- Movie S1: Sunlight driving a soft robot gripper.
- Movie S2: Demonstration of the control scheme.

1 Additional Experimental Information

The spectral power density of thermal electromagnetic radiation from a hot object, such as the sun, is governed by Planck's law of black-body radiation. To estimate the total power per area that a hot object radiates, we can integrate the spectral irradiance profile with respect to the wavelength. Similarly, by multiplying the spectral irradiance by the spectral absorption of the solar absorber film (equation 1), we can estimate the absorbed power per area of the film from the following:

$$P = \int_{\lambda_1}^{\lambda_2} I(\lambda)\alpha_f(\lambda)d\lambda, \tag{1}$$

where λ is the wavelength, $I(\lambda)$ is the spectral irradiance with a unit of W/m^2/nm, and $\alpha_f(\lambda)$ is the spectral absorption profile of the solar absorber film. From the solar spectral irradiance (direct+circumsolar AM 1.5) (*1*), we estimated the solar power density to be 1 kW/m^2. This power density diminishes by a large factor in shadow or indoor environments. To simulate the solar spectrum for indoor experiments, we used Xenon (Xe) lamps. As shown in Figure S1, the irradiance spectrum of a Xe lamp is similar to the solar spectrum. Mercury-Xenon (HgXe) lamps offer a wider bandwidth but are more expensive than Xenon lamps. From the spectral irradiance profile of a 35 W, 75 W, and 150 W Xe lamp (Figure S1), we estimated the output power (at 50 cm) for each to be 3.53 W/m^2, 10.45 W/m^2, and 24.55 W/m^2, respectively. By curve fitting the irradiance vs. input power data, we found the irradiance (at 50 cm) for a 55W Xe lamp to be 6.85 W/m^2. This irradiance is almost 140 times smaller than that of the sun. To enhance the intensity, we used a high beam headlight reflector with a diameter of 80 mm. Assuming the spectral irradiance is measured at r_o (50 cm in our case) and the radius of the reflector is r_r, we can find the amplification gain (G) of the reflector to be:

$$G = \frac{4\pi r_o^2}{\pi r_r^2}. \tag{2}$$

Evaluating equation 2 at the edge of the reflector gives us a gain of about 625, which yields an irradiance of about 4281 W/m^2, more than four times than that of the sun. Radiation from the Xe lamp is in the form of a sphere. When placed in the axis of the reflector (parabolic in this work), the reflector reflects only a portion of the radiation. Therefore, the light is not a perfect collimated beam. Our measurements

show that the light disperses as a function of distance from the reflector, as illustrated in Figure S1C. To better estimate the illumination intensity of the Xe lamp as a function of the distance from the device, we used the data in Figure S1C for the r_r in equation 2. The experiment setup consisted of the device (with the soft robots) held by a clamp and a Xe lamp at a certain distance from the device. As shown in Figure S1D, the vapor pressure increases and pushes the liquid to the soft robots upon excitation.

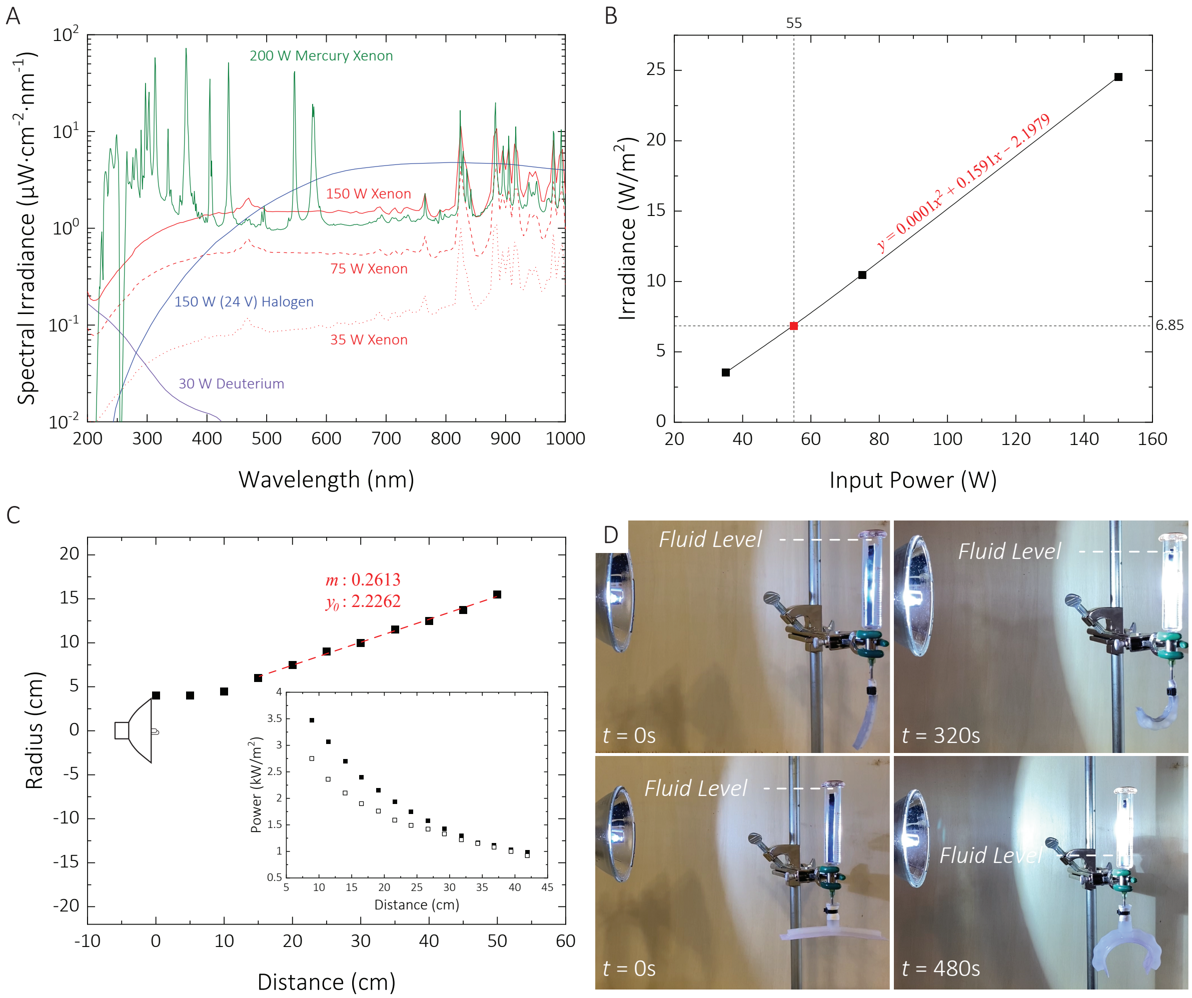

Fig. S 1. (A) Spectral irradiance measured at 50 cm for Xenon (Xe), Mercury-Xenon (HgXe), and Deuterium (D2) continuous-mode lamps (courtesy of Hamamatsu Photonics K.K.). (B) Irradiance vs. input power for a 35W, 75W, and 150W Xenon lamp measured at 50 cm without a reflector. The irradiance for a 55W Xe lamp (red square dot) is estimated from the curve fit to be around 6.85 W/m^2. (C) Illumination radius of a Xe lamp as a function of distance from the reflector. Inset: thermal power measurement with a thermal power meter (Thorlabs S322C). At a short distance, there is about 10% difference between thermal power of the brightest spots (solid points) and darkest spots (hollow points) from the average coming from the beam. At a far distance, the difference from the average decreases to almost 3%. (D) The setup for actuation of the soft robotic prototypes. For the purpose of imaging the setup in one frame, the Xe lamp was placed very close to the device.

2 Control Implementation

We implemented a low-level control scheme in the actuation space to control the pressure of soft robot prototypes. Figure S2 shows the state machine and software flow of the device. Once the sensor and motor are initialized, the device enters the main loop where the pressure is continuously read. Based on the two upper and lower threshold pressure values, the microcontroller decides whether to rotate the motor to a 90° position or a 0° position. For the controller to be activated, the pressure must surpass the upper threshold limit once.

A

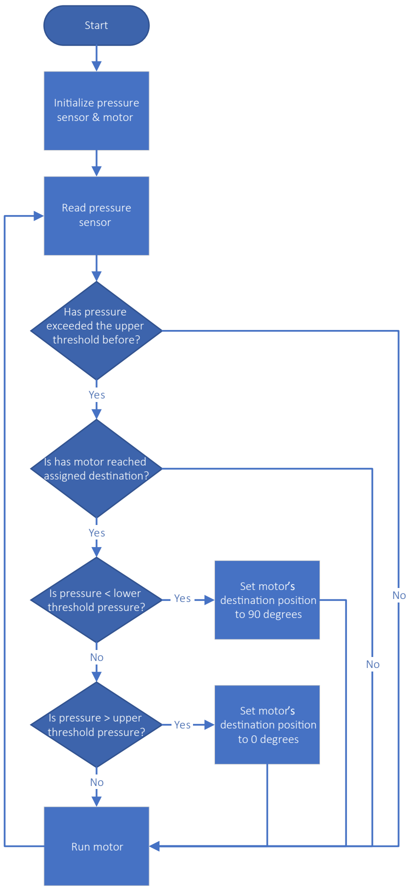

B

```
#include <AccelStepper.h>
// Motor Variables
const int gMotorHalfStep = 8;
const int gMotorPin1 = 7;
const int gMotorPin2 = 6;
const int gMotorPin3 = 5;
const int gMotorPin4 = 4;
// <-------------------------------------
const float gMotorAcceleration = 1000;
const float gMotorMaxSpeed = 400;
const float gMotorCurrentSpeed = 400;
// Pressure Sensor Variables
const float gPressureSensorUnit = 500;
const float gPressureUpperThreshold = 55; // kPa
const float gPressureLowerThreshold = 54; // kPa
bool gStartFlag = false;
// Serial Variables
unsigned long gSerialTimer = 0;
const int gSerialBaudRate = 9600;
const int gPrintTime = 250; // ms
//------------------------------------>
// Initialize with pin sequence IN1-IN3-IN2-IN4 for using the AccelStepper with 28BYJ-48
AccelStepper gMotor(gMotorHalfStep, gMotorPin1, gMotorPin3, gMotorPin2, gMotorPin4);
void setup() {
  Serial.begin(gSerialBaudRate);
  // Config Motor
  gMotor.setMaxSpeed(gMotorMaxSpeed);
  gMotor.setAcceleration(gMotorAcceleration);
  gMotor.setSpeed(gMotorCurrentSpeed);
  gMotor.moveTo(gPressureSensorUnit);
  // Config Pressure Sensor
  analogReference(DEFAULT); // 5V or 3.3V depending on board
}
void loop() {
  // Read pressure
  float pressureRaw = analogRead(A1);
  float pressureKPA = (( pressureRaw / 1024) - 0.04) / 0.00369;
  float readTime = (float)millis() / 1000;
  // Periodic print
  if (millis() > gSerialTimer) {
    Serial.println(readTime);
    Serial.println(pressureRaw);
    Serial.println(pressureKPA);
    gSerialTimer = millis() + gPrintTime;
  }
  // Check thresholds & oscillate
  if (gStartFlag) {
    if (gMotor.distanceToGo() == 0) {
      if (pressureKPA < gPressureLowerThreshold) {
        gMotor.moveTo(gPressureSensorUnit);
      } else if (pressureKPA > gPressureUpperThreshold) {
        gMotor.moveTo(-gPressureSensorUnit);
      }
    }
  } else {
    if (pressureKPA > gPressureUpperThreshold) {
      gStartFlag = true;
    }
  }
  gMotor.run();
}
```

Fig. S 2. (A) State machine flow chart. (B) Source code for the control scheme.

3 Modeling

We developed analytical and numerical models to estimate the transient temperature and pressure inside the light-driven soft robots. The models take the solar irradiance, the system geometry and materials, the fluid thermophysical properties and the ambient environmental conditions as inputs. As outputs, the models gives the time-dependent average fluid temperature, actuator pressure as well as the fluid and gas phases volumes and masses. Our simple 1-D analytical model was compared with a 3-D COMSOL model of the solar-driven actuator, showing good agreement between the two. The use of the 1-D analytical model allows for much faster analysis of the parameters affecting the device performance, while still providing good accuracy of the system's behavior. Details of the 1-D analytical model and 3-D COMSOL are presented next.

3.1 1D analytical model

We can greatly simplify our solar-driven pressure chamber by assuming all its components (e.g., fluid, syringe, sensors, solar absorber, condenser) are at a same temperature T. By assuming an isothermal system, we reduce the complexity of the 3-D system to a simpler 1-D lumped capacitance system, which can more readily be solved for analytically. We first present our general modeling framework for the pressure chamber connected to a soft robotic gripper and then simplify it for the closed the closed pressure chamber. We start by setting a control volume around the pressure chamber (see Fig.S3). This control volume cuts through the connection with the soft robotic grippers, allowing to account for fluid outflow/inflow to/from a soft robotic gripper. Next, we apply the time-dependent first law for an open system to the whole pressure chamber, the ideal gas law to the gas layer and conservation of volume to the fluid and vapor system. The equations are presented below:

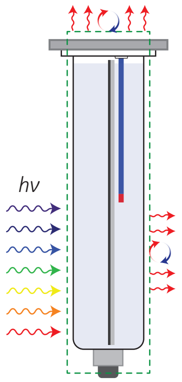

Fig. S 3. Control volume around the solar-drive pressure chamber. The bottom of the syringe can be connected to a soft robot, effectively making our control volume open, or it can be capped to get a closed system.

First law for an open system:

$$\frac{\mathrm{d}E_{syst}}{\mathrm{d}t} = \dot{Q} - \dot{W} + \cancelto{0}{\sum \dot{m}_{in}(u + V^2/2 + gz)_{in}} - \sum \dot{m}_{out}(u + V^2/2 + gz)_{out} \quad (3)$$

Where E_{syst} is the control volume's internal energy, t is time, $\dot{Q}$ is the total thermal energy supplied to the system, $\dot{W}$ is the total work done by the system, $\dot{m}_{in,\ out}$ is the mass flow rate in and out of the system respectively and u, V and gz are the internal, kinetic and potential energies respectively. In the absence of a flow input, we have neglected the mass input to the system $\dot{m}_{in}$. The individual terms in Eq. 3 can be further expanded into:

$$\frac{\mathrm{d}E_{syst}}{\mathrm{d}t} = (\sum m_i c_{p,i})\frac{\mathrm{d}T}{\mathrm{d}t} + c_{p,f}T\frac{\mathrm{d}m_f}{\mathrm{d}t} + (c_{p,g}T + h_{fg})\frac{\mathrm{d}m_g}{\mathrm{d}t} \tag{4}$$

$$\begin{aligned}\dot{Q} &= q_{sun}A_{abs}\tau_{syr}\alpha_{abs} \\ &- (h_{conv,syr}A_{syr} + h_{conv,cond}A_{cond})(T - T_{amb}) \\ &- (\epsilon_{syr}A_{syr} + \epsilon_{cond}A_{cond})(T^4 - T^4_{amb})\end{aligned} \tag{5}$$

$$\dot{W} = \cancelto{0}{\dot{W}_s} + \cancelto{0}{(\dot{m}Pv)_{in}} - (\dot{m}Pv)_{out} = \frac{P}{\rho_f}(\frac{\mathrm{d}m_f}{\mathrm{d}t} + \frac{\mathrm{d}m_g}{\mathrm{d}t}) \tag{6}$$

$$\sum \dot{m}_{out}(u + V^2/2 + gz)_{out} \approx -c_pT(\frac{\mathrm{d}m_f}{\mathrm{d}t} + \frac{\mathrm{d}m_g}{\mathrm{d}t}) \tag{7}$$

where m_i and $c_{p,i}$ are the mass and heat capacity of each components i of the system, h_{fg} is the fluid's latent heat of vaporization, q_{sun} is the solar or light flux, A is the surface area, τ_{syr} is the transmittance of the syringe, α_{abs} is the absorber absorbance, h_{conv} is the convection coefficient with the ambient air (natural convection), ϵ is the surface emissivity, $\dot{W}_s$ is the shaft work done by the system, P is the chamber's pressure, ρ is the density. The subscripts f and g refer to the fluid's liquid and gaseous phases respectively, *abs* refers to the absorber, *syr* refers to the syringe, *cond* refers to the condenser and *amb* refers to the ambient. Kinetic and potential energy are neglected due to the relatively slow velocity of the fluid at the output of the chamber and the small system dimensions.

Ideal gas law (for gas layer only):

$$\frac{\mathrm{d}(PV_g)}{\mathrm{d}t} = \frac{\mathrm{d}(\frac{m_g}{M}RT)}{\mathrm{d}t} \tag{8}$$

$$V_g\frac{\mathrm{d}P}{\mathrm{d}t} + P\frac{\mathrm{d}V_g}{\mathrm{d}t} = \frac{R}{M}(m_g\frac{\mathrm{d}T}{\mathrm{d}t} + T\frac{\mathrm{d}m_g}{\mathrm{d}t}) \tag{9}$$

where V_g is the gaseous phase volume on top of the fluid, R is the gas constant and M is the fluid's molar mass.

Volume Conservation:

$$V_{syr} = V_g + V_f = \frac{m_g}{\rho_g} + \frac{m_f}{\rho_f} \tag{10}$$

To account for the thermophysical properties of the fluid as well as the properties of the soft robotic grippers, the following equations were also used:

$$P(T) = a_1 T^2 + a_2 T + a3 \tag{11}$$

$$V_{gripper}(P) = b_1 e^{-((P-b_2)/b_3)^2} \tag{12}$$

$$dV_{gripper} = dV_g \tag{13}$$

Equation 11 relates the fluid vapor pressure to the fluid temperature assuming a quadratic fit (coefficients a_1, a_2 and a_3) for simplicity, in the temperature range 16°C to 40°C. Equation 12 relates the soft robotic gripper's volume to its internal pressure according to a Gaussian fit (coefficients b_1, b_2 and b_3) of experimentally determined volume vs internal pressure. Equation 13 accounts for the incompressibility of the fluid's liquid state.

We may combine the three main equations (Eqs. 3, 9 and 10) along with the set of other thermophysical properties equations to obtain a set of three differential equations with three unknowns T, m_f and m_g. We can numerically solve for this set of differential equations in MATLAB to obtain the time dependent temperature T and the fluid (m_f) and gas (m_g) masses. A similar approach is used in the case of the closed pressure chamber (no soft robotic gripper attached), although several equations must be changed to account for the change of an open system to a closed system.

$$\dot{W} = 0 \tag{14}$$

$$\sum \dot{m}_{out}(u + V^2/2 + gz)_{out} = 0 \tag{15}$$

$$V_{gripper}(P) = 0 \tag{16}$$

$$\frac{\mathrm{d}m_g}{\mathrm{d}t} = -\frac{\mathrm{d}m_f}{\mathrm{d}t} \tag{17}$$

Similarly to the open system case, the system of equations can be solved for numerically in MATLAB. A summary of the assumptions made in the developing the model are listed below:

- Isothermal system: All components (fluid, gas, glass syringe, sensors and condenser) are at the same temperature T.
- The system's masses and dimensions are taken from the experimental setup.
- The convective heat transfer coefficient (h_{conv}) is estimated from a lumped capacitance model during the cooling of the system from an initial above ambient temperature .
- Kinetic and potential energy are neglected.
- All thermophysical properties of the fluid are taken at 25°C, except for the fluid vapor pressure.
- The fluid vapor pressure is fitted with a quadratic equation in the temperature range 16°C to 40°C for simplicity.
- The robotic gripper volume vs pressure relation is approximated as a Gaussian relation from experimental characterization.
- Diffusion kinetics of the fluid vapor in the gas volume are assumed to be much faster than the evaporation rate (*i.e.*, uniform fluid vapor concentration in the whole gas volume).

3.2 3D numerical model

A 3-D numerical model was also built in COMSOL to validate our simpler analytical model and its assumptions, as well as give additional insights into the performance of the real device. Figure S4 shows the simulation domain corresponding to the pressure chamber in COMSOL. Using symmetry in the device (YZ and XZ planes), only a quarter of it was modeled to save computational time. The model couples heat transfer and laminar fluid flow to account for the effects of natural convection within the fluids (liquid and gas domains). The input solar flux was modeled as a boundary heat flux at the solar absorber. Heat losses from the syringe and the condenser to the ambient were modeled using natural convection and radiative heat transfer. The heat transfer coefficients, the surface emissivity, the ambient temperature and the system's initial temperature (assumed

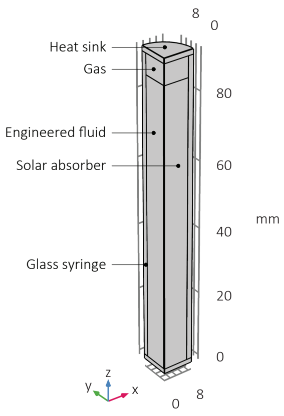

Fig. S 4. Control volume around our solar-driven pressure chamber.

isothermal) were set equal to that of the experiments. The volume of the liquid and gas phases are fixed, assuming negligible fluid evaporation. As such, evaporative heat flux at the liquid/vapor interface was also neglected. The model was solved using a time-dependent solver to capture the transient response of the system. The model's output are the time-dependent temperature profile and the fluid and gas velocity profiles. For comparison with the experiments, the fluid temperature was probed at the center of the syringe as well as 2.15 cm below and above.

References

1. Reference Air Mass 1.5 Spectra, (available at https://www.nrel.gov/grid/solar-resource/spectra-am1.5.html).
